# Methanogens: biochemical background and biotechnological applications

**DOI:** 10.1186/s13568-017-0531-x

**Published:** 2018-01-04

**Authors:** Franziska Enzmann, Florian Mayer, Michael Rother, Dirk Holtmann

**Affiliations:** 1 DECHEMA Research Institute, Industrial Biotechnology, Theodor-Heuss-Allee 25, 60486 Frankfurt am Main, Germany; 2 Technische Universität Dresden, Institut für Mikrobiologie, Zellescher Weg 20b, 01217 Dresden, Germany

**Keywords:** Methanogens, Genetic tools, Biogas, Microbial electrosynthesis, Electroactivity

## Abstract

Since fossil sources for fuel and platform chemicals will become limited in the near future, it is important to develop new concepts for energy supply and production of basic reagents for chemical industry. One alternative to crude oil and fossil natural gas could be the biological conversion of CO_2_ or small organic molecules to methane via methanogenic archaea. This process has been known from biogas plants, but recently, new insights into the methanogenic metabolism, technical optimizations and new technology combinations were gained, which would allow moving beyond the mere conversion of biomass. In biogas plants, steps have been undertaken to increase yield and purity of the biogas, such as addition of hydrogen or metal granulate. Furthermore, the integration of electrodes led to the development of microbial electrosynthesis (MES). The idea behind this technique is to use CO_2_ and electrical power to generate methane via the microbial metabolism. This review summarizes the biochemical and metabolic background of methanogenesis as well as the latest technical applications of methanogens. As a result, it shall give a sufficient overview over the topic to both, biologists and engineers handling biological or bioelectrochemical methanogenesis.

## Introduction

Methanogens are biocatalysts, which have the potential to contribute to a solution for future energy problems by producing methane as storable energy carrier. The very diverse archaeal group of methanogens is characterized by the ability of methane production (Balch et al. 1979). The flammable gas methane is considered to be a suitable future replacement for fossil oil, which is about to be depleted during the next decades (Ren et al. 2008). Methane can be used as a storable energy carrier, as fuel for vehicles, for the production of electricity, or as base chemical for synthesis and many countries do already have well developed natural gas grids (Ren et al. 2008). In terms of the necessary transition from chemical to biological processes, methanotrophic bacteria can use methane as a carbon and energy source to produce biomass, enzymes, PHB or methanol (Strong et al. 2015; Ge et al. 2014). The biological methanation is the main industrial process involving methanogens. These archaea use CO_2_ and H_2_ and/or small organic molecules, such as acetate, formate, and methylamine and convert it to methane. Although the electrochemical production of methane is still more energy efficient than the biological production [below 0.3 kWh/cubic meter of methane (0.16 MPa, Bär et al. 2015)], the biological conversion may be advantageous due to its higher tolerance against impurities (H_2_S and NH_3_) within the educt streams, especially if CO_2_ rich waste gas streams shall be used (Bär et al. 2015). Apart from that, research is going on to increase the energy efficiency of the biological process, so that it might be the preferred way of methane production in the future (Bär et al. 2015). Biological methanation occurs naturally in swamps, digestive systems of animals, oil fields and other environments (Garcia et al. 2000) and is already commonly used in sewage water plants and biogas plants. New applications for methanogens such as electromethanogenesis are on the rise, and yet, there is still a lot of basic research, such as strain characterization and development of basic genetic tools, going on about the very diverse, unique group of methanogens (Blasco-Gómez et al. 2017). This review will summarize important facts about the biological properties and possibilities of genetic modification of methanogenic organisms as well as the latest technical applications. It shall therefore give an overview over the applicability of methanogens and serve as a start-up point for new technical developments.

## Biochemical and microbial background

Methanogens are the only group of microorganisms on earth producing significant amounts of methane. They are unique in terms of metabolism and energy conservation, are widespread in different habitats and show a high diversity in morphology and physiological parameters.

### Phylogeny and habitats of methanogens

For decades known methanogenic archaea belonged exclusively to the phylum *Euryarchaeota*. There, methanogens were classified first into five orders, namely *Methanococcales*, *Methanobacteriales*, *Methanosarcinales*, *Methanomicrobiales* and *Methanopyrales* (Balch et al. 1979; Stadtman and Barker 1951; Kurr et al. 1991). Between the years 2008 and 2012 another two orders of methanogens, namely *Methanocellales* (Sakai et al. 2008) and *Methanomassiliicoccales* (Dridi et al. 2012; Iino et al. 2013), were added to the phylum *Euryarchaeota*. Hydrogenotrophic methanogenesis from H_2_ and CO_2_ is found in almost all methanogenic orders with the exception of the *Methanomassiliicoccales*. Due to its broad distribution it is postulated that this type of methanogenesis is the ancestral form of methane production (Bapteste et al. 2005). Methane formation from acetate, called aceticlastic methanogenesis, can be found only in the order *Methanosarcinales*. In contrast to that, methylotrophic methanogenesis, which is the methane formation from different methylated compounds such as methanol, methylamines or methylated thiols, is found in the orders *Methanomassiliicoccales*, *Methanobacteriales* and *Methanosarcinales*. Extensive recent metagenomic analyses suggested that methanogens may no longer restricted to the *Euryarchaeota*. Two new phyla, namely the *Bathyarchaeota* (Evans et al. 2015) and the *Verstraetearchaeota* (Vanwonterghem et al. 2016) were postulated. Genome sequences from both phyla indicate a methylotrophic methane metabolism in these -as of yet uncultivated- potential methanogens.

Methanogens are a relative diverse group of archaea and can be found in various anoxic habitats (Garcia et al. 2000). For example, they can be cultured from extreme environments such as hydrothermal vents or saline lakes. *Methanocaldococcus jannaschii* was isolated from a white smoker chimney of the East Pacific Rise at a depth of 2600 m (Jones et al. 1983) and *Methanopyrus kandleri* from a black smoker chimney from the Gulf of California in a depth of 2000 m (Kurr et al. 1991). From a saline lake in Egypt the halophilic methanogen *Methanohalophilus zhilinae* was cultured (Mathrani et al. 1988). But methanogens also colonize non-extreme environments. They can be isolated from anoxic soil sediments such as rice fields, peat bogs, marshland or wet lands. For example, *Methanoregula boonei* was obtained from an acidic peat bog (Bräuer et al. 2006, 2011) and several strains of *Methanobacterium* as well as *Methanosarcina mazei* TMA and *Methanobrevibacter arboriphilus* were isolated from rice fields (Asakawa et al. 1995).

Some methanogens can also associate with plants, animals and could be found in the human body. *Methanobacterium arbophilicum* could be isolated from a tree wetwood tissue and uses the H_2_ resulting from pectin and cellulose degradation by *Clostridium butyricum* for methanogenesis (Schink et al. 1981; Zeikus and Henning 1975). From the feces of cattle, horse, sheep and goose the methanogens *Methanobrevibacter thaueri*, *Methanobrevibacter gottschalkii*, *Methanobrevibacter wolinii* and *Methanobrevibacter woesei* have been isolated, respectively (Miller and Lin 2002). In addition, different *Methanobrevibacter* species could be found in the intestinal tract of insects such as termites (Leadbetter and Breznak 1996). Beside the intestinal tract of herbivorous mammals also the rumen contains methanogens. One of the major species here is *Methanobrevibacter ruminantium* (Hook et al. 2010). Methanogenic archaea are also present in the human body. *Methanobrevibacter smithii* and *Methanosphaera stadtmanae* as well as *Methanomassiliicoccus luminyensis* could be detected in human feces (Dridi et al. 2009, 2012; Miller et al. 1982). Further *Methanosarcina* sp., *Methanosphaera* sp. and *Methanobrevibacter oralis* were discovered in human dental plaque (Belay et al. 1988; Ferrari et al. 1994; Robichaux et al. 2003).

Methanogens can be also found in non-natural habitats such as landfills, digesters or biogas plants. There, the microbial community varies with the substrate. In biogas plants, due to hydrolysis of complex polymers to sugars and amino acids, followed by fermentation and acetogenesis, acetate, H_2_ and CO_2_ is produced as substrates for methanogenesis. Therefore, hydrogenotrophic and aceticlastic methanogens are prevalent in mesophilic biogas plants, often dominated by species of *Methanosarcina* (*Methanothrix* at low acetate concentrations) or *Methanoculleus* (Kern et al. 2016b; Karakashev et al. 2005; Lucas et al. 2015; Sundberg et al. 2013). However, under certain conditions syntrophic acetate oxidation may be the dominant path towards methane (Schnürer and Nordberg 2008; Westerholm et al. 2016).

### Diversity of methanogens in morphology and physiological parameters

Methanogens show not only a wide diversity in regard to their habitats but are also highly diverse in terms of morphology, temperature optimum, pH and osmolarity. The shapes of methanogens (only some typical methanogens are mentioned here) can be coccoid as for *Methanococcus*, *Methanosphaera* or *Methanococcoides*, long or short rods as for *Methanobacterium* or *Methanobrevibacter*, or rods in chains as for *Methanopyrus* (Kurr et al. 1991). *Methanoplanus* (Ollivier 1997) has a plate-shaped morphology and *Methanospirillium* (Zeikus and Bowen 1975), as the name says, a spirally shape. *Methanosarcina* (Balch et al. 1979; Bryant and Boone 1987; Kern et al. 2016a; Mah 1980) are irregularly shaped cocci, most often arranged to sarcina cell packages. In addition long filaments formed with rods were observed by species of *Methanothrix* [formerly designated *Methanosaeta* (Kamagata et al. 1992)]. The formation of multicellular aggregates irrespective of the individual cell shape can also occur, like for species of *Methanolobus* (Mochimaru et al. 2009), *Methanosarcina* (Kern et al. 2016a), or *Methanobacterium* (Kern et al. 2015).

The diversity of methanogens is also reflected in the different growth conditions. Many methanogens have a mesophilic temperature spectrum, as, e.g. *Methanosarcina*, *Methanobacterium*, or most *Methanococcus*. However, thermophilic and even hyperthermophilic methanogens are known, like *Methanothermobacter thermautotrophicus* or *M. jannaschii* which grow at temperatures of up to 75 and 86 °C, respectively. Even growth up to 110 °C is possible in hot environments as shown for the hyperthermophilic strain *M. kandleri* (Kurr et al. 1991). In contrast, also cold-loving methanogenic strains could be isolated. One example is the methanol-converting archaeon *Methanolobus psychrophilus*, which grows optimally at 18 °C and shows still metabolic activity at 0 °C (Zhang et al. 2008).

Beside the temperature, salt concentration may also be an important physiological parameter for methanogens. A few methanogens have colonized niches such as saline lakes, which are extreme environments for microorganisms because of their high salinity. Microorganisms living under such salty conditions have to protect themselves from losing water and “salting-out”. Due to the fact that biological membranes are permeable to water, a higher solute concentration outside the cell, as in the case of environments with a high salinity, would drag water out of the cell and would lead to cell death. To prevent the loss of water, and as a countermeasure, microbes increase the cytoplasmatic osmolarity to survive in such salty environments. This can be done in two ways. The first is the synthesis and accumulation of osmoprotectants, also known as compatible solutes, which have a small molecular mass and a high solubility. This has been shown for example for *M. mazei*. At a NaCl concentration of 400 mM the methanogen synthesizes glutamate in response to hypersalinity. At higher salt concentration (800 mM NaCl) *N*-acetyl-β-lysine is synthesized in addition to glutamate (Pflüger et al. 2005, 2003). But *N*-acetyl-β-lysine is not essential for growth and can be also substituted by glutamate and alanine at high salinity (Saum et al. 2009). Moreover it has been also shown that *M. mazei* can take up the osmoprotectant glycine betaine from its environment (Roeßler et al. 2002). The second way to protect the cell from loosing water, and to balance the cytoplasm osmotically with the high salinity of the environment, is an influx of potassium and chloride into the cytoplasm (Oren 2008). This as “high-salt-in strategy” known way may be also used by the recently discovered “Methanonatronarchaeia” (Sorokin et al. 2017). They appear to be extremely halophilic, methyl-reducing methanogens related to the haloarchaea.

Although most (by far) methanogens grow optimally around neutral pH, some, which are halophilic or halotolerant, show also an adaptation to alkaline pH. *Methanocalculus alkaliphilus* grows alkaliphilically with an optimum at pH 9.5 and a moderate salinity up to 2 M of total Na^+^, whereas *Methanosalsum natronophilum* can even tolerate higher salinities, up to 3.5 M of total Na^+^, at the same alkaline pH (Sorokin et al. 2015). Moderately acidic environments can also be inhabited by methanogens as, for example, *Methanoregula booneii*, which was isolated from an acidic peat bog and has an pH optimum for growth of 5.1 (Bräuer et al. 2006, 2011).

### Substrates and metabolism of methanogens

**Fig. 1:**
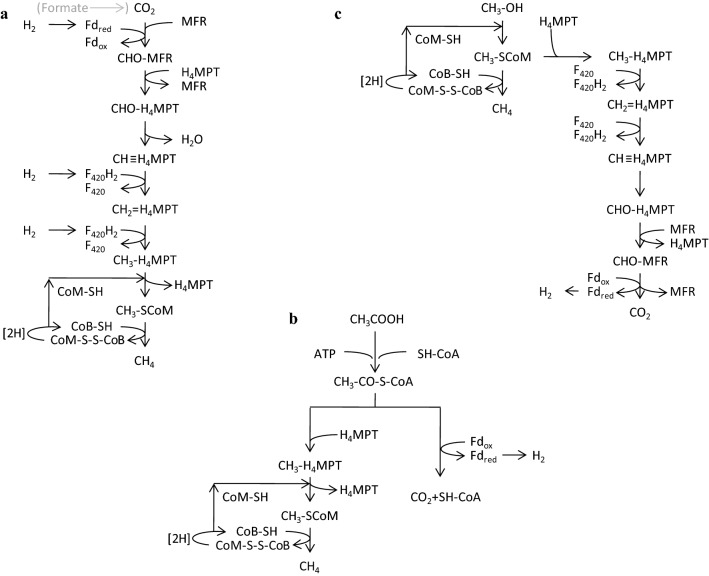
Schematic overview of hydrogenotrophic (**a**), aceticlastic (**b**) and methylotrophic (**c**) methanogenesis. Hydrogenotrophic methanogenesis for Ech-containing methanogens is shown. The methylotrophic methanogenesis from methanol is displayed. Abbreviations are mentioned in the text (Adapted from (Thauer et al. 2008; Welte and Deppenmeier 2014; Welander and Metcalf, 2005))

Methanogens use the substrate CO_2_ and the electron donor H_2_ during hydrogenotrophic methanogenesis. In the first step, CO_2_ is reduced and activated to formyl-methanofuran (Wagner et al. 2016) in which reduced ferredoxin (Fd_red_) is the electron donor for this reaction (Fig. 1).

In the second step the formyl group is transferred to tetrahydromethanopterin (H_4_MTP) obtaining formyl-H_4_MTP. Then the formyl group is dehydrated and reduced to methylene-H_4_MTP and subsequently to methyl-H_4_MTP with reduced F_420_ (F_420_H_2_) as electron donor. The methyl group is then transferred to coenzyme M (HS-CoM). Finally, methyl-CoM is reduced to methane with coenzyme B (HS-CoB) as electron donor. The resulting heterodisulfide (CoM-S-S-CoB) is reduced with H_2_ to recycle the coenzymes (Liu and Whitman 2008; Thauer et al. 2008). It is also important to note that several methanogens can use formate instead of H_2_ as electron source for CO_2_ reduction. There, four formate molecules are first oxidized to CO_2_ by formate dehydrogenase (Fdh) followed by the reduction of one molecule of CO_2_ to methane (Liu and Whitman 2008). Instead of H_2_, a few methanogens can also use alcohols like ethanol or 2-propanol as electron donors (Frimmer and Widdel 1989; Widdel 1986).

Some methanogens can also use carbon monoxide (CO) for methanogenesis. In *Methanosarcina barkeri* and *M. thermautotrophicus* four molecules of CO are oxidized to CO_2_ by CO dehydrogenase (CODH) followed by the reduction of one molecule of CO_2_ to methane with H_2_ as electron donor (Daniels et al. 1977; O’Brien et al. 1984). Thus, growth on H_2_ and CO_2_ is still possible with both methanogens. In contrast, CO metabolism of *Methanosarcina acetivorans* seems to be different. It can also use CO, but is unable to grow on H_2_ and CO_2_ due to the lack of a functioning hydrogenase system. Further, the organism produces high amounts of acetate and formate from CO during methanogenesis (Rother and Metcalf 2004). The genera *Methanosarcina* and *Methanotrix* can use acetate for methane production. In this aceticlastic methanogenesis acetate has to be activated first. It is converted with ATP and coenzyme A (CoA) to acetyl-CoA, which is then split by the CODH/acetyl-CoA synthase complex. The methyl group is transferred to H_4_MTP [which is tetrahydrosarcinapterin (H_4_SPT) in *Methanosarcina*] and further converted to methane like in the CO_2_ reduction pathway. The carbonyl group is oxidized to CO_2_, thus providing the electrons for the methyl group reduction (Welte and Deppenmeier 2014).

The third way of biological methanation is methylotrophic methanogenesis in which methylated substrates as methanol, methylamines or methylated sulfur compounds like methanethiol or dimetyl sulfide, are utilized. Most methylotrophic methanogens belong to the *Methanosarcinales*. In the first step the methyl-group from the methylated substrate is transferred to a corrinoid protein by a substrate-specific methyltransferase (MT1) and subsequently to HS-CoM by another methyltransferase (MT2), thus forming methyl-CoM (Burke and Krzycki 1997). One methyl-CoM is oxidized to CO_2_ (via the hydrogenotrophic pathway in reverse) generating the reducing equivalents to reduce three methyl-CoM to methane and also generating a proton motive force (Timmers et al. 2017; Welte and Deppenmeier 2014).

### Energy conservation in methanogens

In general, methanogens can be divided into two groups according to their mode of energy conservation: methanogens without and with cytochromes (Mayer and Müller 2014; Thauer et al. 2008). Most of the methanogenic archaea do not contain cytochromes. They have a methyl-H_4_MPT:coenzyme M methyltransferase (Mtr) which couples the methyl group transfer to a primary, electrochemical Na^+^ gradient over the membrane (Becher et al. 1992; Gottschalk and Thauer 2001). Furthermore, the H_2_-dependent reduction of CoM-S-S-CoB in cytochrome-free methanogens is catalyzed by a complex consisting of a (methyl viologen-reducing) hydrogenase and heterodisulfide reductase (Mvh-Hdr), which also couples this exergonic process to the concomittant endergonic reduction of oxidized ferredoxin (Fd_ox_) via flavin-based electron bifurcation (Buckel and Thauer 2013). Due to the existence of a Na^+^ binding motif in the *c* subunits of A_1_A_O_ ATP synthases of almost all non-cytochrome containing methanogens (one exception is *Methanosalsum zhilinae*), the established Na^+^ gradient can be used for ATP synthesis (Mayer and Müller 2014; Grüber et al. 2014).

Cytochrome-containing methanogens such as *M. mazei* or *M. barkeri*, also employ Mtr, thus, generating a Na^+^ gradient over the membrane. However, reduction of CoM-S-S-CoB is catalyzed by a membrane-bound heterodisulfide reductase (HdrED), which obtains electrons from reduced methanophenazine (MPhH_2_, functionally analogous to quinoles) via its cytochrome *b* subunit, which is coupled to the generation of a proton motive force. During hydrogenotrophic methanogenesis, a membrane-bound (F_420_ non-reducing) hydrogenase (Vho) oxidizes H_2_ and transfers electrons via cytochrome *b* to oxidized methanophenazine (MPh), again generating a proton motive force. Further, another membranous energy converting hydrogenase, Ech (which is similar to complex I) couples the endergonic reduction of Fd_ox_ with H_2_ to the intrusion of H^+^, i.e., uses the proton motive force (Mayer and Müller 2014; Thauer et al. 2008; Welte and Deppenmeier 2014). Under environmental conditions, e.g. as in a biogas plants, cytochrome-containing *Methanosarcina* are outcompeted by “true” hydrogentrophic methanogens, which produce methane from CO_2_ and H_2_ exclusively.

*Methanosarcina acetivorans* lacks both Vho and Ech. Instead it employs an Rnf complex which is thought to establish a Na^+^ gradient over the membrane by transferring electrons from Fd_red_ (accrued from, e.g., oxidation of CO or oxidation of the carbonyl-group from acetyl-CoA) to MPh. Subsequent electron transport from MPhH_2_ to HdrED again generates a H^+^ gradient (Mayer and Müller 2014; Schlegel et al. 2012b; Welte and Deppenmeier 2014).

The fact that methanogenesis in cytochrome-containing methanogens is coupled to the generation of both a H^+^ and a Na^+^ gradient (Schlegel and Müller 2013) may be also reflected by the ion dependence of their A_1_A_O_ ATP synthases. It has been shown that the A_1_A_O_ ATP synthase from *M. acetivorans* can use both ion gradients (Schlegel et al. 2012a).

During methylotrophic growth of cytochrome-containing methanogens oxidation to CO_2_ involves reduction of cofactor F_420_, which is a 5-deazaflavin derivative. F_420_H_2_ is re-oxidized by F_420_H_2_ dehydrogenase (Fpo), which is a membrane-bound complex (similar to Nuo of *E. coli*) and transfers electrons to MPh, thereby establishing a H^+^ gradient over the membrane in addition to the H^+^ gradients at Hdr and Ech, and the use of the Na^+^ gradient at Mtr (Welte and Deppenmeier 2014).

Analyses of genomes from *Bathyarchaeota* (Evans et al. 2015) and *Verstraetearchaeota* (Vanwonterghem et al. 2016) suggest a methylotrophic methane metabolism for members of these two new phyla. Reduction of the CoM-S-S-CoB in the *Verstraetearchaeota* might be accomplished by the Mvh-Hdr complex which might be coupled to re-oxidation of Fd_red_ by an Ehb or and Fpo-like complex. However, what type of ion gradient (H^+^ and/or Na^+^) might be established over the membrane, is unclear, although H^+^ are predicted to be the coupling ion of the respective A_1_A_O_ ATP synthase (Vanwonterghem et al. 2016). It is obvious that pure culture isolation of *Verstraetearchaeota* is required in order to address the physiology and energy conservation in these potential methanogens.

In the *Bathyarchaeota* energy conservation is even more of a mystery. Two available metagenomes, BA1 and BA2 (proposed to be 91.6 and 93.8% complete, respectively), are missing most of the genes encoding for methanogenic energy conservation. Mtr is incomplete, Fpo as well as an energy-converting hydrogenase (like EhaB, establishing a H^+^ or Na^+^ gradient over the membrane), are missing. In the genome of BA1 only an Ech hydrogenase is encoded. Also, genes encoding for an A_1_A_O_ ATP synthase are absent, which would restrict the organism to ATP synthesis by substrate level phosphorylation (SLP) (Evans et al. 2015).

## Electroactivity of methanogens

### Electron transfer

**Fig. 2:**
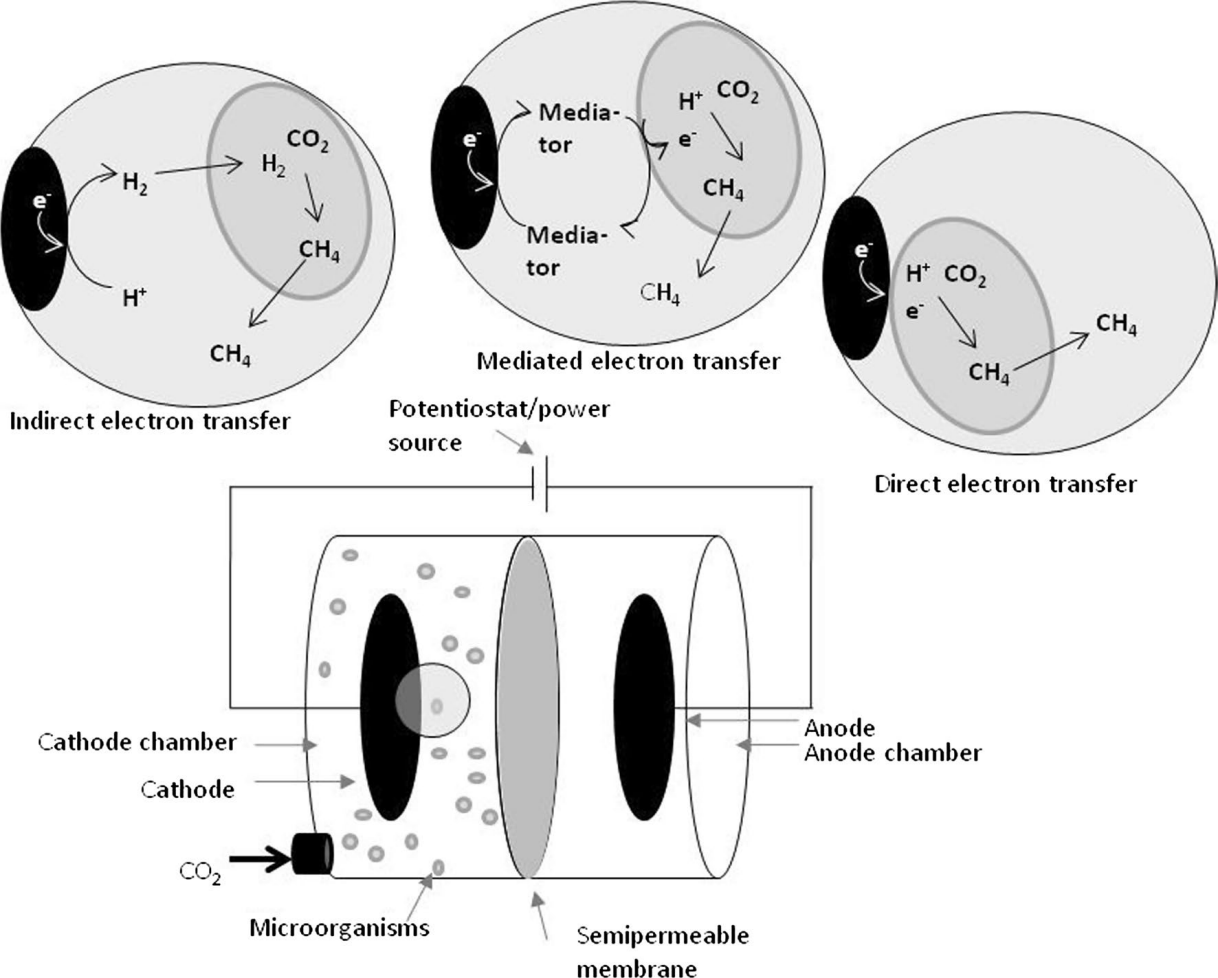
Extracellular electron transfer. Means of electron transfer within a separated, electromethanogenic system at the cathode: indirect electron transfer (IET), mediated electron transfer (MET) and direct electron transfer (DET)

When electrodes are inserted into a reactor with methanogens, these electrodes can eventually be used by the organisms to produce methane. An external potential leads to the electrolysis of water at the anode; oxygen and protons are produced, electrons are transferred to the anode. Otherwise, excess electrons out of metabolic reactions can be transferred to the anode, like it would happen in a microbial fuel cell. The electrons migrate to the cathode through an external circuit. At the cathode surface, the electrons are transferred to the methanogens, which can use them to produce methane. The complete mechanism is not yet elucidated, but mainly, three possibilities are suggested (Fig. 2) (Sydow et al. 2014; Geppert et al. 2016). Probably, more than one of these mechanisms contributes to the electron transfer (Zhen et al. 2015).

One possible way would be the transfer of electrons from the cathode to protons, which have been produced at the anode and migrated through the membrane between anodic and cathodic chamber. Thereby, hydrogen is produced at the cathode, which is then consumed by the methanogens. This indirect electron transfer (IET) would allow the production of methane out of hydrogen and CO_2_ (Villano et al. 2010). As an example, IET was observed in *M. thermautotrophicus* (Hara et al. 2013). It has also been shown that some *Methanococcus maripaludis* secrete hydrogenases and probably formate dehydrogenases which catalyze the formation of hydrogen and formate directly at the electrode surface; the produced hydrogen and formate is then metabolized by the cells (Deutzmann et al. 2015). This has to be seen as an indirect electron transfer, since the cells were not directly attached to the electrode; from the experimental results, it may be mistaken for a direct electron transport, since the abiotically (without catalyzing hydrogenases) produced amounts of hydrogen and formate cannot explain the amount of methane produced (Deutzmann et al. 2015).

Another possibility suggests that mediator molecules could accept the electrons at the cathode surface, shuttle it to the methanogens and donate it to the microorganisms. This mediated electron transfer (MET) would imply that the methanogens take up electrons, protons and CO_2_ to form methane (Choi and Sang 2016). Flavins, phenazines or quinones can serve as mediator, either naturally secreted by the organisms or added to the reaction medium (Sydow et al. 2014; Patil et al. 2012). A natural secretion of mediators with a redox potential suitable for microbial electrosynthesis (should be < − 0.4 V vs. SHE) has not been observed yet (Sydow et al. 2014). In methanogens, MET could be performed by using neutral red as an electron shuttle (Park et al. 1999).

The third option would be the direct electron transfer (DET) from the cathode surface to the methanogens, e.g. via surface proteins or conductive filaments (so-called nanowires). To generate methane, the microorganisms would use electrons, protons and CO_2_ (Cheng et al. 2009). Several studies suggest that direct electron transfer indeed occurs in methanogens (Zhen et al. 2016; Lohner et al. 2014). For a hydrogenase-deficient strain of *M. maripaludis* hydrogenase-independent electron uptake was demonstrated (Lohner et al. 2014), ruling out IET.

In a mixed microbial consortium, direct interspecies electron transfer (DIET) is another possible way of electron transfer. There, one microbial strain takes up electrons at the cathode surface and transfers it to another strain. This may happen, e.g. via conductive filaments (Gorby et al. 2006). It has been reported that this (syntrophic) electron transfer can be very specific between two species, e.g. based on conductive filaments between *M. thermautotrophicus* and *Pelotomaculum thermopropionicum* (Gorby et al. 2006) or between *M. barkeri* and *Geobacter metallireducens* (Rotaru et al. 2014). Apart from this direct interspecies electron transfer, an interspecies hydrogen transfer can occur. Here, one organisms takes up electrons, produces hydrogen as an intermediate and transfers them to a second organism that forms another product. An example is the defined co-culture between the iron-corroding, sulfate-reducing bacterium ‘Desulfopila corrodens’ IS4 (former name: *Desulfobacterium corrodens*) for electron uptake and *M. maripaludis* for methane production (Deutzmann and Spormann 2017).

### Electroactive methanogens

Up to date, most investigations on electromethanogenesis have been carried out with mixed cultures, e.g. from wastewater treatment plants, biogas plants or microbial fuel cells. In technical applications, mixed cultures might be more resistant against environmental stress (Babanova et al. 2017), but it is hard to conclude how many and which methanogenic strains are electroactive by themselves. From analysis of the mixed cultures studied, it can be concluded which methanogens are enriched and are therefore likely to be electroactive, although mixed culture experiments cannot replace pure culture studies to prove electroactivity. These are for example *Methanobacterium palustre* (Cheng et al. 2009; Batlle-Vilanova et al. 2015; Jiang et al. 2014), *Methanosarcina thermophila* (Sasaki et al. 2013), *M. thermautotrophicus* (Sasaki et al. 2013; Fu et al. 2015), *Methanoculleus thermophilus* (Sasaki et al. 2013), *Methanobacterium formicicum* (Sasaki et al. 2013), *M. maripaludis* (Deutzmann and Spormann 2017), *Methanococcus aeolicus* (Feng et al. 2015), *M. mazei* (Feng et al. 2015), *M. arboriphilus* (Jiang et al. 2014), *Methanocorpusculum parvum* (Jiang et al. 2014) and *Methanocorpusculum bavaricum* (Kobayashi et al. 2013). In other studies, the dominant methanogenic organism has not been defined exactly or not explicitly mentioned (Batlle-Vilanova et al. 2015; Bo et al. 2014; Zhen et al. 2015). Only few studies have been carried out with pure cultures instead of mixed cultures, so these methanogens are the only ones that are certainly electroactive. To mention are *M. thermautotrophicus* (Hara et al. 2013), and a *Methanobacterium*-like strain IM1 (Beese-Vasbender et al. 2015).

Yet, just a minority of methanogenic strains has been tested for electroactivity, mostly under similar growth conditions. Unfortunately, no specific marker for electroactivity has been found yet (Koch and Harnisch 2016). It is therefore possible that more electroactive methanogens, active even under more extreme conditions, exist.

## Genetic tools for methanogens

Many properties of a (model) organisms are unraveled by biochemical and physiological analysis; however where neither of the two lead to satisfactory insight, genetic analysis is often desirable. Furthermore, the accessibility of an organism relevant for applied purposes to genetic manipulating opens the possibility for targeted engineering by removal of -or amendment with- metabolic or regulatory functions. The principal requirements for such a system are sufficiently efficient methods to (a) isolate clonal populations (e.g., via plating on solid media), to (b) transfer genetic material (i.e., protocols for transformation, transduction, or conjugation), and to (c) link the transfer of the genetic material to an identifiable (i.e., screenable or selectable) phenotype (e.g., conferred by marker genes).

The biochemistry of the methanogenic pathway, the trace elements required, as well as the nature and structure of unusual (C1-carrying) cofactors involved has been elucidated using various *Methanobacterium* strains (some of them now reclassified as *Methanothermobacter*). Therefore, it was a logical next step to develop genetic systems for these models. Plating of *Methanothermobacter* on solid media could be achieved which allowed isolation (and consequently characterization) of randomly induced mutations (Harris and Pinn 1985; Hummel and Böck 1985). However, this species could not be developed into model organisms for genetic analysis because the transfer of genetic material is too inefficient (Worrell et al. 1988). Furthermore, the use of selectable phenotypes was (and still is) restricted because antibiotics (in conjuncture with the respective genes conferring resistance) commonly used in bacterial genetics are ineffective in archaea due to the differences in the target structures (e.g., cell wall, ribosomes). Therefore, the establishing of an antibiotic selectable marker (the *pac* gene from *Streptomyces alboniger* conferring resistance to puromycin) in *Methanococcus voltae* (Gernhardt et al. 1990) was key to the development of gene exchange systems in methanogens. Another feature of *Methanococcus*, which facilitated method development, is absence of pseudomurein from its cell wall; instead, the organism is surrounded by a proteinacous surface (S-) layer that can be removed with polyethylene glycol (PEG), resulting in protoplasts, which apparently can take up DNA. Combined with its comparably robust and fast growth on H_2_ + CO_2_ *Methanococcus* species, most prominently *M. maripaludis*, prevailed as the genetic model for hydrogenotrophic methanogens, for which many useful genetic tools have been developed (Table 1, and see Sarmiento et al. 2011 for a review).

**Table 1:** Genetic elements used for manipulation of methanogens

Element(s)	Type	Use/phenotype	Organism^a^	References
(Counter-) selectable markers				
*pac*	Resistance gene	Puromycin resistance	*Methanococcus*, *Methanosarcina*	Gernhardt et al. (1990) and Metcalf et al. (1997)
APH3’II	Resistance gene	Neomycin resistance	*M.* *maripaludis*	Argyle et al. (1996)
*apH*-*2b*	Resistance gene	Neomycin resistance	*M. mazei* ^*b*^	Mondorf et al. (2012)
*ileS3*	Resistance gene	Pseudomonic acid resistance	*M.* *acetivorans*	Boccazzi et al. (2000)
*serC*	Biosynthesis gene	Auxotrophic marker	*M.* *barkeri*	Metcalf et al. (1996)
*proC*	Biosynthesis gene	Auxotrophic marker	*M.* *acetivorans*	Pritchett et al. (2004)
*hpt*	Purine salvage gene	Counter-selectable marker (8-aza-2,6-diaminopurine)	*Methanosarcina*	Pritchett et al. (2004)
*upt*	Purine salvage gene	Counter-selectable marker (6-aza-uracil)	*M.* *maripaludis*	Moore and Leigh (2005)
Controlling gene expression				
*PhmvA*	Promoter	Constitutive gene expression	*Methanococcus*	Beneke et al. (1995)
*Psl*	Promoter	Constitutive gene expression	*Methanococcus*	Sun and Klein (2004)
*PmcrB/Tmcr*	Promoter/terminator	Constitutive gene expression	*Methanococcus*, *Methanosarcina*	Gernhardt et al. (1990) and Mūller et al. (1985)
*PmcrB(tetO)*/TetR	Promoter/repressor	Tetracycline-dependent gene de-repression	*Methanosarcina*	Guss et al. (2008)
*PmtaCB1*	Promoter	Methanol-dependent gene induction	*M.* *acetivorans*	Rother et al. (2005)
*PmtbC*	Promoter	Methylamine-dependent gene induction	*M. mazei*	Mondorf et al. (2012)
*Pnif*/NrpR	Promoter/repressor	N-dependent gene repression	*M.* *maripaludis*	Cohen-Kupiec et al. (1997 and Lie and Leigh (2003)
tc-RS4	Riboswitch	Tetracycline-dependent gene repression	*M.* *acetivorans*	Demolli et al. (2014)
Random mutagenesis				
*Himar1*/Tnp	Insect transposon/transposase	Random transposon mutagenesis	*M.* *acetivorans*, *M.* *maripaludis*	Zhang et al. (2000) and Sattler et al. (2013)
*Tn5/*Tnp	Transposon/transposase	Random transposon mutagenesis	*M.* *maripaludis*	Porat and Whitman (2009)
Reporter genes				
*lacZ*	Reporter gene	Quantification of gene expression/promoter function	*M.* *maripaludis*	Gardner and Whitman (1999)
*uidA*	Reporter gene	Quantification of gene expression/promoter function	*M. voltae*, *Methanosarcina*	Beneke et al. (1995) and Pritchett et al. (2004)
*bla*	Reporter gene	Quantification of gene expression/promoter function	*M.* *acetivorans*	Demolli et al. (2014)
Facilitating recombination/mutagenesis				
$\Phi {\text{C}}31$ *int* *attB/P*	Phage recombination system	Site specific chromosomal integration	*Methanosarcina*	Guss et al. (2008)
*flp*/FRT	Yeast recombination system	Marker rescue	*Methanosarcina*, *Methanococcus*	Welander and Metcalf (2008) and Hohn et al. (2011)
Cas9	Nuclease	Targeted genome editing	*M.* *acetivorans*	Nayak and Metcalf (2017)

^a^If genus is given, the element is effective in various model species

^b^The construct used does not confer resistance to neomycin in *M.* *acetivorans* (Matschiavelli and Rother, unpublished)

For methylotrophic methanogens containing cytochromes (*Methanosarcina* species) genetic methodology was initially developed on existing tools. PEG-mediated transformation was reported to be ineffective [but later shown to require only modest modifications of the existing protocol (Oelgeschläger and Rother 2009)], but cationic liposomes could be used to transform *Methanosarcina* species (Metcalf et al. 1997). Like in *Methanococcus*, presence of an S-layer and availability of an autonomously replicating cryptic plasmid [pC2A in *Methanosarcina* (Metcalf et al. 1997) and pURB500 in *Methanococcus* (Tumbula et al. 1997)], which could be engineered into shuttle vectors also replicating in *E. coli*, made (heterologous) gene expression comparably easy. Markerless insertion and/or deletion of genes was achieved by establishing counter-selective markers, which are used to remove “unwanted” DNA from the chromosome (Pritchett et al. 2004; Moore and Leigh 2005).

Chromosomal integration and deletion of DNA in methanogens, which can be rather inefficient, mostly relies on homologous recombination requiring sequences of substantial length (500–1000 bp) to be cloned. Thus, establishing site-specific recombination by engineering a *Streptomyces* phage recombination system ($\Phi {\text{C31}}$) to integrate DNA into (Guss et al. 2008) -and the yeast Flp/FRT system to remove DNA from- the chromosome (Welander and Metcalf 2008), was a major progress for the genetic manipulation of *Methanosarcina*. The recent successful -and highly efficient- application of the CRISPR/Cas9-system from *Streptococcus pyogenes* (Doudna and Charpentier 2014) for gene deletion and insertion in *M. acetivorans* (Nayak and Metcalf 2017) holds the promise of an even easier way to genetically manipulate these important organisms. Most tools (Table 1) developed for one methanoarchaeal model organism can usually be adapted for use in another, as exemplified by exploiting the insect transposable element *Himar1* together with its transposase for random mutagenesis in *Methanosarcina* (Zhang et al. 2000) and, later, in *Methanococcus* (Sattler et al. 2013). Thus, any progress made will likely be useful for all other model systems.

Although it might not be possible to use genetically modified methanogens in the established methanogenic processes like biogasproduction or wastewater treatment, new genetic tools are necessary to guarantee the progress in methanogenic research. It will get clear in the next sections that modified methanogens can be used for bioproduction.

## Applications of methanogens

Methanogenic archaea are a very diverse group and some strains can grow under extreme conditions, like extremely high or low temperatures, high osmolarities or pH values. Therefore, the development and optimization of industrially applicable processes making use of methanogens is desirable. This is not only true in terms of methane production as a technical relevant fuel (Ravichandran et al. 2015), but also for other products and applications.

### Hydrogen production

It has been observed that several methanogenic strains can also produce hydrogen (Valentine et al. 2000; Goyal et al. 2016). This can happen if the amount of available hydrogen is limited (sub-nanomolar), so that the methanogens seem to start metabolic hydrogen production instead of hydrogen consumption; it has turned out that not methane, but formate and possibly other metabolites can be the source of H_2_; this cannot be seen as reverse methanogenesis (Valentine et al. 2000; Lupa et al. 2008). The hydrogen production observed by Valentine et al. reached 0.25 μmol/mg cell dry mass for *Methanothermobacter marburgensis*, 0.23 μmol/mg cell dry mass for *Methanosaeta thermophila* strain CALS-1 and 0.21 μmol/mg cell dry mass for *M. barkeri* strain 227 (Valentine et al. 2000). Several strains of *M. maripaludis* produced 1.4 μmol/mg of hydrogen per milligram of cell dry mass, out of formate (Lupa et al. 2008). This application is still restricted to the lab scale (Valentine et al. 2000) and to create a reasonable process, genetic engineering would have to be done to increase the hydrogen yield (Goyal et al. 2016). It is assumed that the hydrogenases present in methanogens are the enzymes catalyzing the hydrogen production (Valentine et al. 2000). A possible way to increase the hydrogen yield could therefore be the detection of the relevant hydrogenase and afterwards overexpressing it.

### Biotechnological production by genetically modified methanogens

During recent years, genetic tools for methanogens have been improved, opening a new field of research on these important microorganisms. As a first step, the product spectra of methanogens could be increased. For example, it has been possible to modify *M. maripaludis* to produce geraniol instead of methane from CO_2_ + H_2_ or from formate (Lyu et al. 2016). Apart from allowing different products, it has also been possible to broaden the substrate range. As an example, the introduction of a bacterial esterase allowed *M. acetivorans* to grow on methyl-esters (like methyl acetate and methyl propionate, Lessner et al. 2010). In wild type methanogens, “trace methane oxidation” (i.e., “reverse methanogensis”) has been reported to occur during net methane production (Timmers et al. 2017). It has been possible to use this effect for acetate production: Heterologous expression in *M. acetivorans* of genes encoding methyl-CoM reductase from anaerobic methanotrophic archaea (ANME-1) resulted in a strain that converted methane to acetate three times faster than the parental strain (Soo et al. 2016). Also, additional expression of the gene encoding 3-hydroxybutyryl-CoA dehydrogenase (Hbd) from *Clostridium acetobutylicum* resulted in formation of l-lactate (0.59 g/g methane) from methane with acetate as intermediate, possibly by Hbd exhibiting lactate dehydrogenase activity in the heterologous host (McAnulty et al. 2017). Thus, the principal possibility might exist to engineer *M. acetivorans* for industrial production. However, as both conversion rates and product yields were low and for neither case the conversion stoichiometries reported, the applicability of such a system remains in question. The same holds true for the production of other high value products like amino acids or vitamins with methanogens, and due to their slow growth, a technical application is not yet developed (Schiraldi et al. 2002). But since there is continuous progress in the development of genetic tools for methanogens, as described above, it is thinkable that new processes with heterologeous methanogens will emerge during the next years.

### Methane from oil and coal beds

Nearly two-thirds of the fossil oil remains within the oil fields if using conventional production methods (Gieg et al. 2008). It was observed that the residual oil can be converted to natural gas by a methanogenic consortium, which was added to the oil field (Gieg et al. 2008). The consortium used was gained from subsurface sediments and could be enriched with crude oil. *Methanosaeta* spec. was the dominant archaeon in the enrichment, which also contained syntrophic sulfate-reducing bacteria, *Clostridiales*, *Bacteroidetes* and *Chloroflexi*. The consortium was added to samples of petroliferous cores from different oilfields, with residual oil saturation of the sandstone grains of approximately 30–40%. Methane could be produced with yields of up to 3.14 mmol/g crude oil (Gieg et al. 2008). Apart from oil fields, also oil sands tailing ponds or other oil–water emulsions could be treated that way (Voordouw 2011). But since costs for natural gas remain relatively low, whilst those for crude oil are significantly higher, this approach remains experimental due to lack of benefit (Voordouw 2011). A natural source of methane is coal bed methane. It has been discovered that about 40% of this methane are produced by microbial consortia containing methanogens (e.g. *Methanosarcinales*); the substrates for this production are methoxylated aromatic compounds within the coal beds (Mayumi et al. 2016). It was recently discovered that pure cultures of *Methermicoccus shengliensis* can produce up to 10.8 μM/(g coal) methane (Mayumi et al. 2016). Coal bed methane is already industrially used; it might be possible to use *M. shengliensis* for methane production from other sedimentary organic material (Mayumi et al. 2016).

### Biogas production from organic matter

The main technical application of methanogens is the production of biogas by digestion of organic substrates. It is estimated that up to 25% of the bioenergy used in Europe could be produced using the biogas process until 2020 (Holm-Nielsen et al. 2009). Digestion of organic matter can be seen as a four-stage process. During the first step (hydrolysis), complex organic matter (proteins, polysaccharides, lipids) is hydrolyzed by exo-enzymes to oligo- and monomers (amino acids, sugars, long chain fatty acids), which can be taken up by microorganisms (Vavilin et al. 2008). The second step, fermentation or acidogenesis, leads to an oxidation of the compounds formed during hydrolysis to typical fermentation products like butyrate, propionate, acetate, formate, ethanol, H_2_ and CO_2_. Acetogenesis represent the third step, where the fermentation products are oxidized, mostly to acetate and CO_2_ with the concomitant formation of H_2_ (Batstone et al. 2002). However, this process is only sufficiently exergonic for the organisms if the H_2_ partial pressure is kept very low (McInerney et al. 2008). This requires the fourth step, methanogenesis, where acetate (and methylated compounds) and CO_2_ and H_2_ is converted to methane by the methanogens. This implicates that a syntrophic consortium of microorganisms is always needed, whereas the exact composition of this consortium can not only change over time, but also vary between different reactors (Solli et al. 2014). Depending on the microbial community and the type of methanogens within, this process can be carried out in psychrophilic, mesophilic or thermophilic temperature range (Vanegas and Bartlett 2013). For stable biogas production, hydrolysis, acidogenesis, acetogenesis and methanogenesis have to run within the digester in balanced reaction rates to prevent the overacidification of the reactor by surplus protons. However, the microorganisms responsible for these different steps often have different optimal growth conditions, so it is crucial that conditions are maintained, which favor all steps (Niu et al. 2015). Therefore, careful control of process parameters like temperature (Vanegas and Bartlett 2013), hydraulic retention time (Rincón et al. 2008), pH (Lay et al. 1997) and ammonia concentration (Karakashev et al. 2005) are necessary. Apart from that, the biogas yield and the process operation and conditions strongly depend on the type of substrate used (Niu et al. 2015). It was for example observed that the methanogenic consortium, which is strongly depending on the substrate type, is usually dominated by *Methanosaetaceae* in digesters with sludge as substrate, while solid waste digesters operated with manure explained in the following section usually host a majority of *Methanosarcinaceae* (Karakashev et al. 2005). In both cases, methanogens that can metabolize acetate (see also “Substrates and metabolism of methanogens” section) are preferred in biogas systems, compared to those feeding on hydrogen and CO_2_. Apart from the substrate type itself, a differentiation is made between wet and dry fermentation, whereby the more common wet fermentation includes up to 10% of solids in the substrate, and the dry fermentation between 15 and 35% (Stolze et al. 2015).

#### Treatment of sewage water

The treatment of sewage water by anaerobic digestion does not only lead to biogas production but also to clean water. Using a methanogenic process to convert the organic matter within wastewater to biogas reduces the amount of sludge to be disposed, lowers its pathogenic potential and usually needs less additional energy than aerobic processes, since biogas as energy fuel is produced and no energy intense aeration is necessary (Martin et al. 2011). Apart from that, the greenhouse gas emission of the anerobic process is lower when treating high strength waste waters, although no greenhouse gas savings could be detected for low strength sewage water (Cakir and Stenstrom 2005).

A commonly used system for the anaerobic treatment of wastewater is the upflow anaerobic sludge blanket (UASB) reactor; wastewater enters the reactor from the bottom and flows to an outlet in the upper part of the reactor. Sludge particles out of the waste water agglomerate and form a sludge blanket, which has to be passed by the incoming wastewater. In this zone, the methanogenic consortium digests organic material and produces biogas, which leaves the reactor at its top. Since the solubility of methane in water is low compared to that of CO_2_, the holdup of methane within the water is negligible (Sander 2015). The contact between organic material and microorganisms is sufficient for efficient methane production due to the sludge blanket, thus allowing higher loading rates than in other reactor types. The system only requires a low energy input, but needs a long start-up phase of several months, until the sludge blanket has fully established (Rajeshwari et al. 2000). To overcome long start up phases, continuously stirred tank reactors can be used, but here, organic loading rates are about tenfold lower than in the UASB reactor (Rajeshwari et al. 2000). It is important to consider the type of sewage water (e.g. from breweries, paper mills, oil mills, dairy production or other) when estimating the biogas yield of digestion. Different organic loads or different substrate composition lead not only to fluctuating amounts of biogas, but also to changes of the biogas composition (reviewed by Tabatabaei et al. 2010). Instead of treating sewage water itself via anaerobic digestion, it is also possible to purify the water by aerobic processes and anaerobically digest the remaining sewage sludge (Van Lier et al. 2008). Usually, a pretreatment of the sludge can increase the biogas yield. This can for example, but not only, be an alkaline pretreatment, ozonation, ultrasonic pretreatment or electric pulses to increase the biodegradability of the sludge (Wonglertarak and Wichitsathian 2014; Bougrier et al. 2007; Rittmann et al. 2008; for a recent review see: Neumann et al. 2016).

#### Treatment of solids

The largest amount of biodegradable waste for biogas production can be obtained from the agricultural sector. This includes animal manure and slurry from the production of pig, poultry, fish and cattle (Holm-Nielsen et al. 2009). The treatment of agricultural wastes like animal manure with methanogenic consortia is not only beneficial in terms of the biogas produced. It also reduces odors and pathogens and is therefore increasing the fertilizer qualities of the manure (Sahlström 2003). The process of biogas formation does not necessarily have to be coupled to waste treatment. Biogas plants can also be operated with energy crops cultured for the biogas production, like sugar beet or maize silage (Demirel and Scherer 2008; Lebuhn et al. 2008). Another possibility is the anaerobic digestion of microalgae, which lowers the necessary cultivation area (Mussgnug et al. 2010). Especially if energy crops without addition of manure are digested, it can be necessary to add micronutrients to ensure optimum growth conditions (Choong et al. 2016). It is also important to consider that lignocellulosic materials are not fully convertible without pretreatment, which leads to lower methane yields (Zheng et al. 2014). Table 2 shows production yields for different solid substrates.

**Table 2:** Biogas production from organic wastes

Substrate	Biogas (ml/gVS)	Methane (ml/gVS)	Methane content (%)	References
Food waste	784	518	66.1	Liu et al. (2009)
Green waste	631	357	56.5	Liu et al. (2009)
Bovine manure	150	40	46.5	Fantozzi and Buratti (2009)
Chicken manure	220	110	66.6	Fantozzi and Buratti (2009)
Pig manure	412	216	52	Amon et al. (2006)
Sugar beet	730	387	53	Weiland (2010)
Grass	211	150	71	Yu et al. (2002)
Maize	560	291	52	Weiland (2010)
Microalgae (*Chlamydomonas reinhardtii*)	784	518	66.1	Mussgnug et al. (2010)
Microalgae (*Arthrospira platensis*)	631	357	56.5	Mussgnug et al. (2010)

*VS* volatile solids

**Fig. 3:**
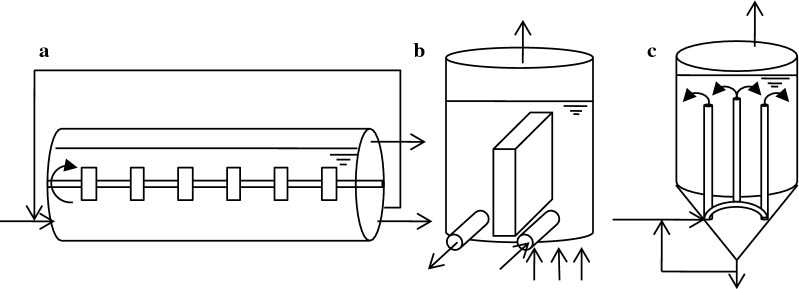
Plug flow digesters for biogas production. **a** “Kompogas” reactor. Horizontal plug flow reactor. Additional mixing by axial mixer. Increased process condition stability by partial effluent recycling. Gas outlet on top of the outlet side. 23–28% total solids. **b** Valorga reactor. Substrate entry at the bottom; plug flow over a vertical barrier to the outlet. Additional mixing by biogas injection at the bottom. 25–35% total solid content. **c** Dranco reactor. Substrate entry wit partial effluent recycling at the bottom, upward flow through substrate pipes. Downward plug flow to outlet. 30–40% total solids (Li et al. 2011; Nizami and Murphy 2010)

A crucial aspect of the biogas process is the design of the anaerobic digester (Nizami and Murphy 2010). There are several digester types for the anaerobic digestion of wastewater. For the digestion of solids, biogas plants are usually designed as continuously stirred tank reactors (CSTRs). Even though this might be the easiest and cheapest way of biogas production, it turned out that the efficiency can be increased by using a serial system. Here, two CSTRs are used; biogas yield was increased by a longer overall retention time (Boe and Angelidaki 2009; Kaparaju et al. 2009). Instead of CSTRs, plug-flow systems have been invented by different companies to perform continuous processes (Fig. 3); in a serial digestion, they would usually be taken for the first stage (Weiland 2010). Another possibility is the use of a batch process, especially for substrates with low water contents, for example in a garage type fermenter (Li et al. 2011; Nizami and Murphy 2010).

#### Micro biogas systems

**Fig. 4:**
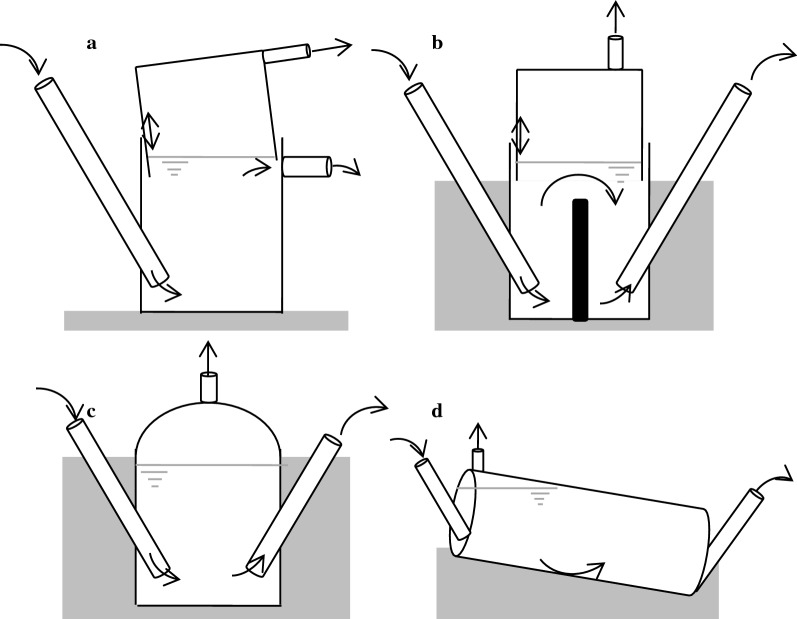
Micro biogas systems. **a** Arti biogas (India). Material two plastic water tanks (working volume of 1 m^3^). Substrate mainly kitchen waste. Disadvantage of gas losses of up to 20% (Voegeli et al. 2009). **b** Floating cover (India). Material bricks and metal cover. Top rises when gas is produced. Substrate mainly pig and cow manure (Bond and Templeton 2011). **c** Fixed dome (China). Material bricks and clay. Substrate mainly pig and cow manure (Plöchl and Heiermann 2006). **d** Plug flow. Material affordable plastic foils (Bond and Templeton 2011)

An interesting application of the biogas process is the use of micro biogas plants in developing countries. These plants of up to 10 m^3^ can be operated using domestic organic waste or feces, while the produced gas can be used directly for heating and cooking. There are also attempts to convert the biogas out of those digesters with volumes of up to 10 m^3^ to electricity, which might be valuable in rural areas (Plöchl and Heiermann 2006). These reactors are particularly popular in China and India and programs to equip households with biogas energy are supported by the government (Bond and Templeton 2011). Domestic biogas plants are especially beneficial in warm regions (e.g. Africa around the equator, South-East Asia) with sufficient water available. In general, 3 types of digesters are used, which are the fixed dome, the floating cover digester, which was further developed to the ARTI biogas system, and the plug flow (or tube) system (Fig. 4).

Although micro biogas systems might not solve the energy problems in developing countries, and the investment costs may not be covered without governmental subsidy, some positive impacts of this technology can be observed. The deforestation in rural areas decreases since wood is not needed for heating, at the same time risks caused by open fire in closed buildings are minimized by the use of a biogas driven stove. The amount of pathogens in the substrate (waste and feces) is decreased, so that it can be reused as fertilizer (Bond and Templeton 2011). Therefore, microbiogas systems are an important contribution to the development of third world countries and a use of the biogasprocess not standing in conflict to food-production, since organic waste is the main substrate.

#### Biogas composition and process optimizations

The composition of biogas does not only include methane, but also up to 40% CO_2_, water, hydrogen sulfide and other trace gases. Biogas is usually flammable due to the high yield of methane (40–75%), but for the use in engines or for injection into the natural gas grid it has to be purified and upgraded in methane content. This leads to higher calorific values of the biogas and avoids the presence of corrosive gases like hydrogen sulfide, which could cause damages to engines and pipes if remaining in the biogas (Ryckebosch et al. 2011). There are several upgrading techniques, which take place after digestion (extensively reviewed by Bauer et al. 2013). Process optimization can influence the biogas composition already during the process, lowering the costs of after-process purification. Numerous investigations on improvement of the biogas process have been undertaken, either to increase the overall amount of biogas, or to increase the methane content of the biogas.

It has turned out that careful pretreatment of the organic substrates leads to higher percentages of methane in the biogas. Several pretreatment methods such as chopping, alkali treatment and thermal treatment are reviewed in Andriani et al. (2014). From a biotechnological point of view, biological pretreatment of substrate is especially interesting. Biological pretreatment can increase the biogas production; this method was described by Zhong et al. (2011) which led to a 33% increase of biogas production (Zhong et al. 2011). The substrates were exposed to a microbial agent including yeasts, celluleutic bacteria and lactic acid bacteria, which degraded the substrate before the actual start of the anaerobic digestion. A reduction in lignin, cellulose and hemicelluloses content could be observed after 15 days of pretreatment. The following anaerobic digestion showed an increase of biogas yield and methane content (Zhong et al. 2011). Apart from the pretreatment of the single substrates, a mixture of different substrates (co-digestion) or a backmixing of digester effluent can lead to a better performance of the system (Weiland 2010; Sosnowski et al. 2003). Co-digestions can be carried out with mixtures of manure and energy plants or sewage slug and solid wastes and increase the methane production because of stabilizing the C:N ratio within the digester (Ward et al. 2008; Sosnowski et al. 2003). Another optimization method is addition of inorganic particles to the fermentation medium. Addition of nanoparticles of zero-valent iron could enhance the methane production by 28% (Carpenter et al. 2015). An increase in biogas formation could also be observed with magnetic iron oxide particles (Abdelsalam et al. 2017). Other particles include charcoal, silica and mineral salts were investigated (reviewed by Yadvika et al. 2004). The improvement in biogas yield could be due to aggregation of bacteria and methanogens around the particles, leading to a lower washout and higher culture densities; it is also possible that metal particles release electrons to the surrounding medium, which can be used for methane formation, but the exact mechanism remains unclear (Yadvika et al. 2004; Carpenter et al. 2015).

**Fig. 5:**
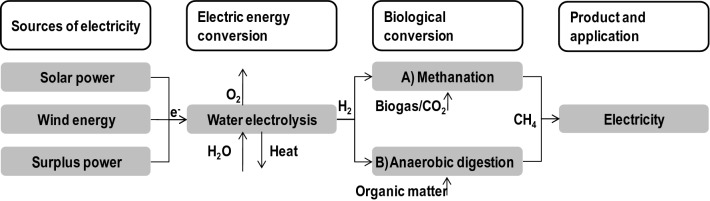
Increasing methane yield by hydrogen addition. H_2_ is produced via water electrolyses and (A) fed into the second reactor for the conversion of CO_2_ into methane, or (B) feed directly to the anaerobic digester for in situ methane production

One promising method for biological biogas upgrading in methane content is the conversion of the residual CO_2_ to additional methane using hydrogenothrophic methanogens, which are capable of producing methane solely out of CO_2_ and H_2_ (Bassani et al. 2015). H_2_ can either be injected into the anaerobic digester (Luo et al. 2012), or H_2_ and biogas can be mixed in a second reactor containing methanogens (Bassani et al. 2015; Luo and Angelidaki 2012) (Fig. 5). If introducing hydrogen to the anaerobic digester, there may be a shift within the methanogenic community: acetoclastic methanogens decrease, while hydrogenotrophic methanogens (especially *Methanoculleus*) are enriched; also, hydrolyzing and acidifying bacteria decrease, while synthrophic bacteria producing acetate increase (see also “Substrates and metabolism of methanogens” section; Bassani et al. 2015). Technical concepts for the integration of H_2_ into existing biogas plants and effective new means of process control are necessary to make this process commercially attractive. Therefore, experiments have to be carried out under industrial conditions, i.e. under fluctuating substrate compositions, in reactors with zones of different substrate concentrations, changing microbial consortium and different pressure zones according to a larger reactor height; these conditions will usually not appear in lab scale, unless they are particularly tested.

H_2_ is usually produced by water electrolysis, a process in which electricity is used to split water and generate oxygen and hydrogen. To couple water electrolysis to anaerobic methanogenesis and provide a constant level of H_2_ within the digester, methanogenic bioelectrochemical systems were invented.

### Methanogenic bioelectrochemical systems

The successful increase of methane production by iron addition leads to the conclusion that methanogens may use inorganic surfaces to boost their metabolism by exchange of electrons with the inorganic material (Carpenter et al. 2015). On the other hand, hydrogen addition could also increase the methane output of a biogas plant. A methanogenic bioelectrochemical system (BES) combines these two improvements for increased methane production (Koch et al. 2015). Here, electrodes are introduced into the reaction medium and an external potential is applied. Methanogens can now either interact directly with the electrode surface to gain electrons (Cheng et al. 2009), and/or hydrogen can be produced at the cathode, which can then be consumed by the methanogens to produce methane (Geppert et al. 2016). The whole process belongs to the field of microbial electrosynthesis (MES), which includes processes that convert a substrate into a desired organic product by using microorganisms and electrical current (Schröder et al. 2015; Lovley 2012; Holtmann et al. 2014). The advantage of a BES system compared to the external production of hydrogen is that short time storage and gassing in of the hardly soluble hydrogen can be avoided (Butler and Lovley 2016).

**Fig. 6:**
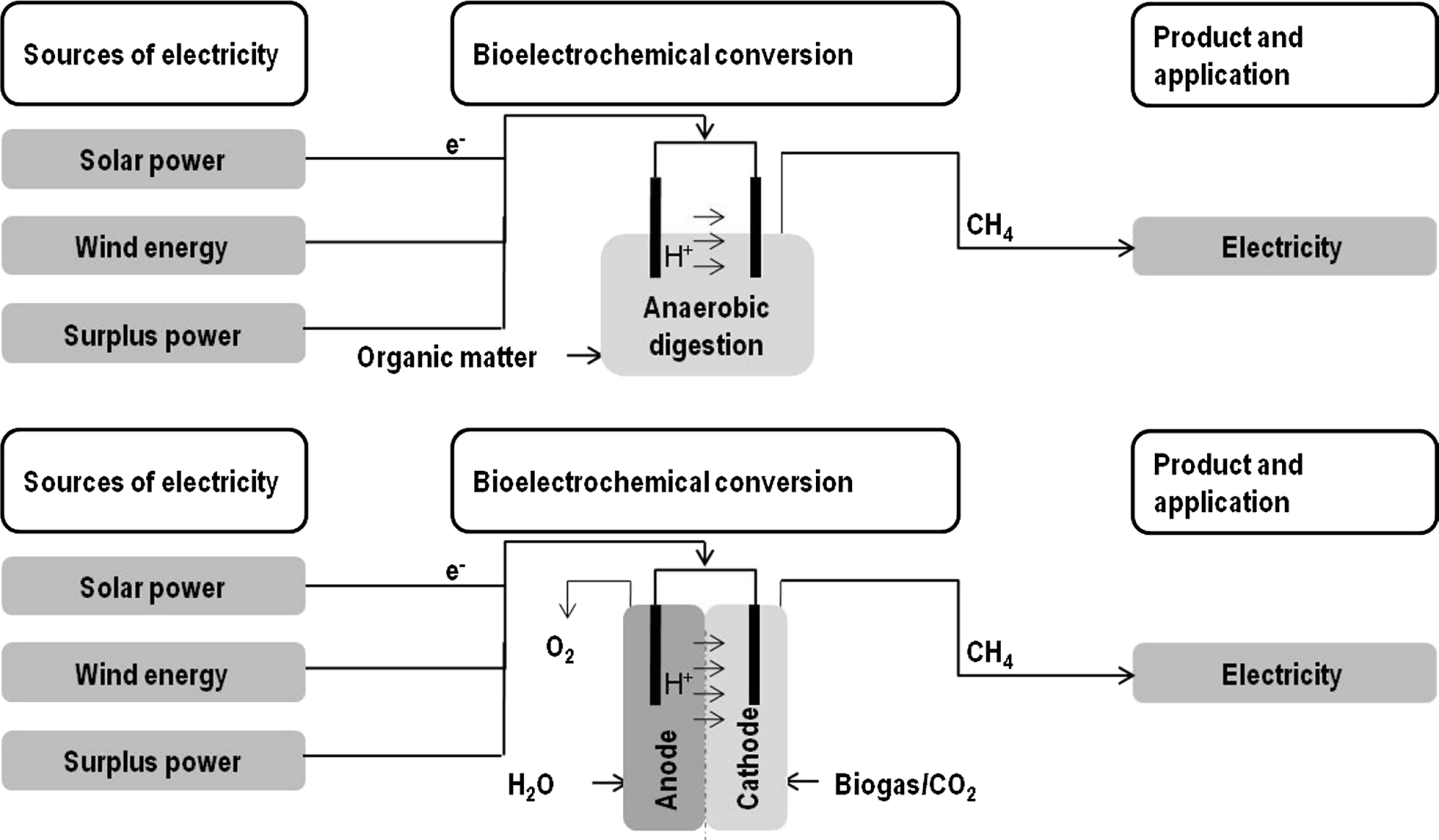
Increasing methane yield by electrode integration. Top: integration of electrodes into the anaerobic digester; bottom: biogas upgrading in an external, separated MES system fed with CO_2_ and electricity

Notably, the electrode material and size, the membrane material and size and the applied voltage strongly influences the performance of electromethanogenesis, (see Babanova et al. 2017; Krieg et al. 2014; Ribot-Llobet et al. 2013; Siegert et al. 2014 for reviews), but “optimal” conditions for microbial growth and production have not yet been found (Blasco-Gómez et al. 2017). Investigations of this (relatively new) technology have been mostly carried out in lab scale so far, with very few pilot scale approaches (for hydrogen production with methane as side product, see Cusick et al. 2011). Yet, no scale up concept or even well characterized reactor concept exists for electromethanogenesis, whereas various types of bioelectrochemical reactors have been designed (reviewed in Geppert et al. 2016; Krieg et al. 2014; Kadier et al. 2016). Two general modes of integrating electrochemistry into the methanogenic process can be distinguished: first, the electrodes can be integrated into the anaerobic digestion of sewage water or other organic wastes, and secondly, the methanogenic BES can be placed into a second reactor as a stand-alone-process, fed with CO_2_, but without additional organic substrates (Fig. 6).

#### Integration of electrodes into waste and wastewater treatment

To enhance the production of biogas and increase biogas purity, electrodes can be inserted into the anaerobic digester for in situ biogas upgrading. CO_2_, which is produced during the digestion of organic matter, can be converted to methane at the electrodes without an additional reactor (Bo et al. 2014). Therefore, the biogas production can be performed during wastewater treatment (Guo et al. 2017) or sewage sludge treatment (Guo et al. 2013) as well as in a mere biogas producing process (Gajaraj et al. 2017). The methane content within the biogas reached up to 98.1% during the digestion of activated sludge and acetate (Bo et al. 2014). It has been shown that the integration of electrodes alters the microbial consortium within the plant, while it is also possible to use adapted consortia, e.g. for psychrophilic temperature ranges (Koch et al. 2015; Bo et al. 2014; Liu et al. 2016). To achieve a reasonable process, ways of electrode integration into existing treatment plants need to be established.

#### Bioelectrochemical systems fed with CO_2_

Methanogenic microbial electrosynthesis can also be carried out in a second reactor, which is equipped with electrodes and fed with CO_2_ or a gas containing CO_2_. Gas streams rich in CO_2_ can be biogas, syngas, or industrial flue gas. The CO_2_ contained is often considered a waste component of these gas streams, and since it is also a greenhouse gas, the conversion of CO_2_ to more useful chemicals is desirable (Dürre and Eikmanns 2015; Geppert et al. 2016). The conversion of CO_2_ by methanogens takes place at the cathode of the system. Since anodic processes like oxygen generation or acid production could inhibit the methanogens, the process can be carried out in a two-chamber system, were anode and cathode chamber are separated by a proton-exchange membrane, which allows the transfer of protons from anode to cathode chamber; this is necessary to allow electrical current in the system and maintain the pH within the cathode chamber (Dykstra and Pavlostathis 2017; Cheng et al. 2009). In this system, it is possible to use a pure methanogenic culture (Beese-Vasbender et al. 2015) or an enriched methanogenic consortium (Dykstra and Pavlostathis 2017) at the cathode, while the anode chamber can be abiotic (water electrolysis) or biotic (degradation of organic matter) (Dykstra and Pavlostathis 2017).

As mentioned, the bioelectrochemical methanogensis is currently still a lab-scale application. To gain a economical technical process, concepts for process characterization and control, reactor balancing, and scale up of reactors have to be developed. To create further progress in this field and also in bioelectrochemical applications, genetic tools might be necessary to create methanogens with higher electron uptake rates, e.g. via the integration of (more) cytochromes into the membrane or the heterologeous secretion of electron shuttles.

## Conclusions

Methanogens are interesting organisms, both from a biological, as well as for a technological, point of view. Research of the last years made it clear that this unique group of microbes is far from being fully understood. During the last years, several reviews on biological aspects of methanogens (Borrel et al. 2016; Goyal et al. 2016), on natural methanogenesis (e.g. Park and Liang 2016; Bao et al. 2016) or on single technical applications, eventually in combination with the very specific biology within the process (Biogas: Wang et al. 2017; Braguglia et al. 2017; Koo et al. 2017; Biogas upgrading and optimization: Neumann et al. 2016; Choong et al. 2016; Romero-Güiza et al. 2016; Bioelectromethanation: Blasco-Gómez et al. 2017; Geppert et al. 2016) have been published. All these review articles are rather specialized to one single aspect of methanogens. This review combines all these aspects, including a review of recently developed tools, to give an overview over the whole field of methanogenic research. Therefore, it makes it possible to understand challenges in industrial applications by giving the biological basics and helps to imagine applications for results from basic research in industry. Industry mainly focused on the production of biogas with methanogens, but other applications, especially when considering electroactivity of methanogens, seem feasible. Newly developed genetic tools for methanogens are useful to design a wider product spectrum, which raises the technical relevance of methanogens. However, most processes possible with methanogens are still not economically feasible, since their strict requirement for anaerobic conditions raises the investment costs and their slow growth leads to long process times. It would be desirable to have further comparable knowledge of the efficiency of different methanogenic strains in terms of space time yield and conversion rates under industrially relevant conditions, for example by performing pure culture studies with fluctuating substrate composition, fluctuating pH and under different substrate concentrations. A major problem here remains the comparability of published data about methanogenic performance in biogas plants as well as in electrochemical systems, since studies have been carried out under various conditions. For some applications, especially microbial electrosynthesis, more research of the methanogenic community and comparisons between pure and mixed cultures have to be done to increase methane yields. Still, process optimization, like the use of CO_2_-rich waste gas streams as substrates and intelligent process integration will favor methanogenic processes beyond waste treatment in the future. Scale-up of reactors, e.g., for electromethanogenesis or biogas-upgrading, are a major task for process engineers, while genetic engineering may pave the way to produce higher value products from waste CO_2_ employing methanogens.

## Abbreviations

BES: bioelectrochemical system
CoA: coenzyme A
CoB: coenzyme B
CoM: coenzyme M
CODH: CO dehydrogenase
CSTRs: continuously stirred tank reactors
DET: direct electron transfer
DIET: direct interspecies electron transfer
Ech: energy converting hydrogenase
Fd: ferredoxin
Fd_ox_: oxidized ferredoxin
Fd_red_: reduced ferredoxin
Fdh: formate dehydrogenase
Fpo: F_420_H_2_ dehydrogenase
Hbd: 3-hydroxybutyryl-CoA dehydrogenase
Hdr: heterodisulfide reductase
H_4_MTP: tetrahydromethanopterin
H_4_SPT: tetrahydrosarcinapterin
IET: indirect electron transfer
MES: microbial electrosynthesis
MET: mediated electron transfer
MFR: methanofuran
MPh: methanophenazine
MPhH_2_: reduced methanophenazine
Mtr: methyl-H_4_MPT:coenzyme M methyltransferase
Mvh-Hdr: (methyl viologen-reducing) hydrogenase and heterodisulfide reductase
PHB: polyhydroxybutyric acid
SHE: standard hydrogen electrode
SLP: substrate level phosphorylation
UASB: upflow anaerobic sludge blanket
Vho: (F_420_ non-reducing) hydrogenase
